# Putative Roles for Peptidylarginine Deiminases in COVID-19

**DOI:** 10.3390/ijms21134662

**Published:** 2020-06-30

**Authors:** Elif Damla Arisan, Pinar Uysal-Onganer, Sigrun Lange

**Affiliations:** 1 Gebze Technical University, Institute of Biotechnology, Gebze, 41400 Kocaeli, Turkey; d.arisan@gtu.edu.tr; 2 Cancer Research Group, School of Life Sciences, University of Westminster, London W1W 6UW, UK; p.onganer@westminster.ac.uk; 3 Tissue Architecture and Regeneration Research Group, School of Life Sciences, University of Westminster, London W1W 6UW, UK

**Keywords:** SARS-CoV-2, COVID-19, peptidylarginine deiminases (PADs), protein deimination, extracellular vesicles (EVs), immunity, comorbidities, NETosis, anti-viral

## Abstract

Peptidylarginine deiminases (PADs) are a family of calcium-regulated enzymes that are phylogenetically conserved and cause post-translational deimination/citrullination, contributing to protein moonlighting in health and disease. PADs are implicated in a range of inflammatory and autoimmune conditions, in the regulation of extracellular vesicle (EV) release, and their roles in infection and immunomodulation are known to some extent, including in viral infections. In the current study we describe putative roles for PADs in COVID-19, based on in silico analysis of BioProject transcriptome data (PRJNA615032 BioProject), including lung biopsies from healthy volunteers and SARS-CoV-2-infected patients, as well as SARS-CoV-2-infected, and mock human bronchial epithelial NHBE and adenocarcinoma alveolar basal epithelial A549 cell lines. In addition, BioProject Data PRJNA631753, analysing patients tissue biopsy data (n = 5), was utilised. We report a high individual variation observed for all PADI isozymes in the patients’ tissue biopsies, including lung, in response to SARS-CoV-2 infection, while PADI2 and PADI4 mRNA showed most variability in lung tissue specifically. The other tissues assessed were heart, kidney, marrow, bowel, jejunum, skin and fat, which all varied with respect to mRNA levels for the different PADI isozymes. In vitro lung epithelial and adenocarcinoma alveolar cell models revealed that PADI1, PADI2 and PADI4 mRNA levels were elevated, but PADI3 and PADI6 mRNA levels were reduced in SARS-CoV-2-infected NHBE cells. In A549 cells, PADI2 mRNA was elevated, PADI3 and PADI6 mRNA was downregulated, and no effect was observed on the PADI4 or PADI6 mRNA levels in infected cells, compared with control mock cells. Our findings indicate a link between PADI expression changes, including modulation of PADI2 and PADI4, particularly in lung tissue, in response to SARS-CoV-2 infection. PADI isozyme 1–6 expression in other organ biopsies also reveals putative links to COVID-19 symptoms, including vascular, cardiac and cutaneous responses, kidney injury and stroke. KEGG and GO pathway analysis furthermore identified links between PADs and inflammatory pathways, in particular between PAD4 and viral infections, as well as identifying links for PADs with a range of comorbidities. The analysis presented here highlights roles for PADs in-host responses to SARS-CoV-2, and their potential as therapeutic targets in COVID-19.

## 1. Introduction

SARS-CoV-2 infections mainly target the lung, compared with other viral infections which begin with upper respiratory tract symptoms. Importantly, SARS-CoV-2 infection does not follow regular viral lower respiratory infection pathways, and furthermore shows additional comorbidities such as vascular responses [1,2,3,4], cardiovascular and cardiomyopathy [5,6,7], gastrointestinal involvement [8], kidney injury [9,10], cutaneous manifestations [11] and stroke [12]. Structural proteins of SARS-CoV-2 include the matrix (M) protein, nucleocapsid (N) protein (for virus entrance and spread), small envelope (E) protein, as well as the multifaceted spike (S) glycoprotein [13,14]. Due to fundamental knowledge gaps with regard to specific molecular pathways for viral-host interactions, the lack of successful current therapeutic intervention strategies, and the variation of host immune responses to the virus, investigations into associated immune-mediated mechanisms are urgently needed.

Peptidylarginine deiminases (PADs) are calcium-dependent, phylogenetically conserved enzymes that cause the post-translational conversion of arginine to citrulline, in an irreversible manner, in target proteins. Deimination can lead to structural, as well as functional, changes of target proteins, including cytoskeletal, nuclear, mitochondrial and cytoplasmic proteins, leading to, amongst other things, the generation of neo-epitopes, as well as changes in gene regulation [15,16,17,18,19,20]. The protein structures most susceptible to deimination are beta-sheets and disordered proteins [16], while deimination can also contribute to protein moonlighting, allowing one polypeptide to carry out multifaceted functions. Such deimination/citrullination-mediated moonlighting may therefore contribute to the protein’s diverse functions in a range of physiological and pathological scenarios [21,22]. In humans, five PADI genes, clustered in a single locus at 1p35-36, encode five PAD isozymes (PAD1, PAD2, PAD3, PAD4, PAD6), with tissue-specific expression and deimination activity linked to a range of pathologies and inflammatory responses. This includes cancer, chronic, autoimmune and neurodegenerative diseases [19,20,23], CNS injury [24,25,26] and ageing [27,28]. Importantly, roles for PADs have been described in viral [29,30,31] and other pathogenic infections, including sepsis, endotoxemia [29,32,33,34,35,36,37,38] and antibiotic resistance [39]. The roles for PADs in anti-viral responses include PAD-mediated neutrophil extracellular trap formation (NETosis), for example, in respiratory syncytial virus disease [40]. Recent work in comparative animal models has furthermore described roles for PADs in innate, adaptive and mucosal immunity [41,42,43,44,45,46,47,48]. Roles for PADs in lung disease have been described due to pollution [49], in bronchial and alveolar mucosa in response to harmful stimuli [50], as well as in lung inflammation and cystic fibrosis [51,52,53].

PADs have also been identified as key regulators of cellular extracellular vesicle (EV) release [23,54,55,56], which is a central factor in many pathologies, including infections [20,57,58,59,60]. EV-mediated responses have therefore received a great deal of interest in COVID-19 [61,62,63], particularly seeing as EV signatures can be useful biomarkers [64,65]. PAD are conserved throughout the phylogenetic tree [15,41], and both PADs and their deiminated protein products are detected in a range of taxa [24,25,41,43,45,46,47,48,66,67], including in bacteria [39], fungi [68] and parasites [69], with some pathogens using their PAD homologues for immune evasion [70]. This places PADs as important factors in immune modulation throughout phylogeny and, due to the fact that coronaviruses are zoonotic viruses [71], the role for PADs in host–pathogen interactions may therefore be of considerable importance for zoonosis as well.

Due to the multifaceted roles of PADs in inflammatory diseases and infection, as well as its association with a range of chronic conditions [72], some of which are also associated with COVID-19 [73], and PAD-mediated roles in skin [74], contribution to NETosis and mucosal immunity [43,44], this study aimed at identifying putative roles for PADs in host–pathogen responses to SARS-CoV-2. For this purpose, we performed an in silico analysis based on recently published BioProject Data, assessing PRJNA615032 and PRJNA631753, involving tissue biopsies from SARS-CoV-2 patients as well as in vitro cell line-based studies. Our reported findings highlight the potential of PADs as targets for novel therapeutic strategies to regulate inappropriate inflammatory responses, as well as for their roles in comorbidities, and for modulating host–pathogen interactions in COVID-19.

## 2. Results

### 2.1. Summary of PAD Expression in BioProject Data PRJNA631753

The understanding of the relationship between SARS-CoV-2 lung infection and the severity of pulmonary disease is currently limited. In the BioProject, viral RNA has been visualised using in situ hybridisation, and the immune infiltrate has furthermore been assessed using immunohistochemistry and the performing of total RNA sequencing on five COVID-19 positive patients. Patients who had high levels of viral RNA did show hyaline membranes, as assessed by histology, alongside low T-cell numbers and extensive pneumocyte loss. Enrichment was also observed in the interferon gene signature. Patients who had lower levels of viral RNA displayed lower T-cell and CD8 cell numbers, and furthermore showed enrichment for genes associated with fibrosis. Overall study design of the BioProject was as follows: Autopsy samples obtained from SARS-CoV-2-infected diseased patients were collected for total analysis for RNA-seq. Then, viral load and immune response assessment was carried out (details provided at: https://www.ncbi.nlm.nih.gov/bioproject/).

#### PADI 1-6 Isozyme Expression is Differently Regulated in SARS-CoV-2-Infected Tissue

**Figure 1:**
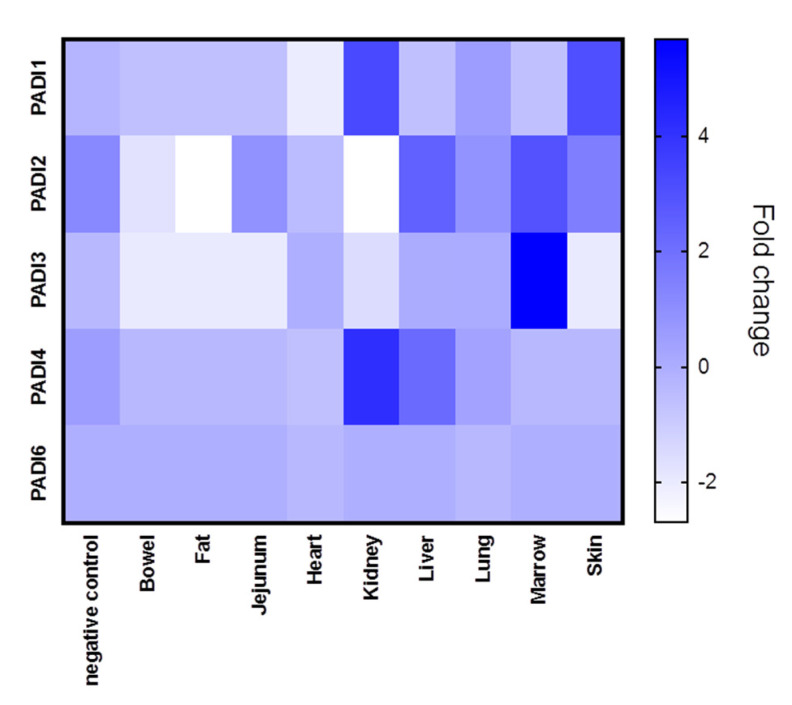
PRJNA631753 samples were analysed according to fold-change for PADI1, PADI2, PADI3, PADI4 and PADI6 mRNA levels. Average data from different tissue types are presented with a heat map. Lung and heart samples were from 5 different cases, alongside normal lung samples (“negative control”), with at least 3 replicate biopsy results. The bowel, kidney and liver data were from 2 cases. The data given for fat, jejunum, marrow and skin tissues were from one case. Heat map metric is given as blue bar between normalised mRNA levels.

The different tissue biopsies from the five patients, compared with normal lung biopsies, are shown in Figure 1, where averages in PADI expression (mRNA) for the five cases are represented for each tissue type (see also Supplementary Figures S1–S5 for PADI 1-6 expression in individual cases). In all figures provided, PAD gene data has been obtained from the normalised gene expression profile using the Rosalind bioinformatics tool.

Tissue expression for the different PADI isozymes (PADI 1,2,3,4 and 6, respectively), revealed differences in both tissue-specific expression, as well as between COVID-19 and control cases (lung tissue only), and individual variation was also shown.

**Figure 2:**
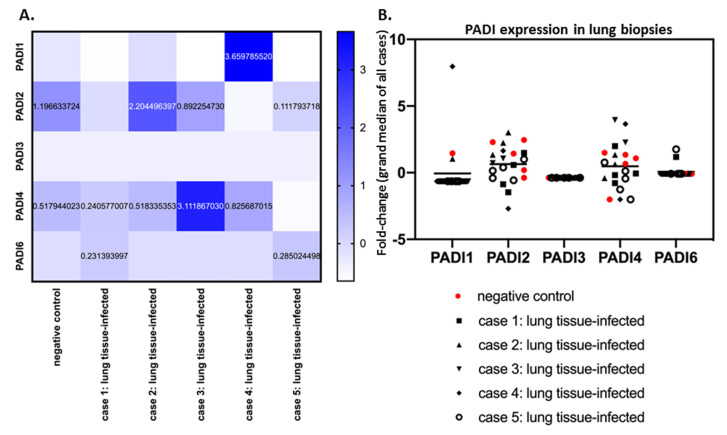
PADI 1-6 isozyme expression levels in SARS-CoV-2-infected lung biopsies, and control lung tissue. (**A**) PRJNA631753 Bioproject data were analysed according to fold change for PADI1, PADI2, PADI3, PADI4 and PADI6 mRNA levels. Average data from control and SARS-CoV-2-infected lung tissue types are presented with a heat map. (**B**) The fold change for mRNA expression levels for each PADI is presented for each case and replicate via grand median distribution of data. Negative control (normal lung samples) and SARS-CoV-2 lung samples were from 5 different cases, with at least 3 replicate biopsy results for lung tissue.

PADI1 was overall elevated in the skin and kidney (Figure 1), with individual variation observed, specifically in the skin of case five, in the kidney and lung of case 4, and in the lung of case 2 (Supplementary Figure S1). PADI2 showed elevation in the liver and marrow (Figure 1) as well as the lung of Covid-19 case 2 (Supplementary Figure S2). PADI3 was found to be overall elevated in the marrow (Figure 1), the kidney and the lung of one COVID-19 case only (Supplementary Figure S3). PADI4 was found elevated in SARS-CoV-2-infected kidneys and livers (Figure 1), but was also elevated in the marrow (case 5) as well as the lung tissue (case 1, 2) and heart (case 1) (Supplementary Figure S4). For PADI6, expression was found to be elevated in the lung tissue of two cases (case 1 and case 3), while no elevation was observed in other tissues (Supplementary Figure S5) (Figure 1). See summary of all tissues in Figure 1, and a separate figure for lung tissue specifically (Figure 2).

When comparing lung tissues only from SARS-CoV-2-infected patients (five cases) with negative control lung tissue, a considerable individual variation for the different PADIs was observed (Figure 2A,B). PADI2 and PADI4 showed the most variability in mRNA levels, both in control tissue and SARS-CoV-2 cases (Figure 2B). Compared with control lung tissue, PADI2 was up to 1.84-fold increased (case 2), but downregulated in other cases. Overall, PADI4 showed the highest differences, compared with control lung tissue, with up-regulation from 1.57 to 6.10-fold, but negligible change in one case, and downregulation in two, compared with normal lung (Figure 2A). PADI3 did not change in lung tissue in response to SARS-CoV-2 infection. Elevation for PADI1 was observed in one COVID-19 case specifically (7-fold elevation; Figure 2A,B) and some elevation in PAD6 was seen in the lungs of two cases, but no effect in the other three cases (Figure 2B).

### 2.2. PADI Expression is Differently Regulated in SARS-CoV-2-Infected Human Bronchial Epithelial Cells (NHBE) and Adenocarcinoma Human Alveolar Basal Epithelial Cells (A549) Cell Lines

**Figure 3:**
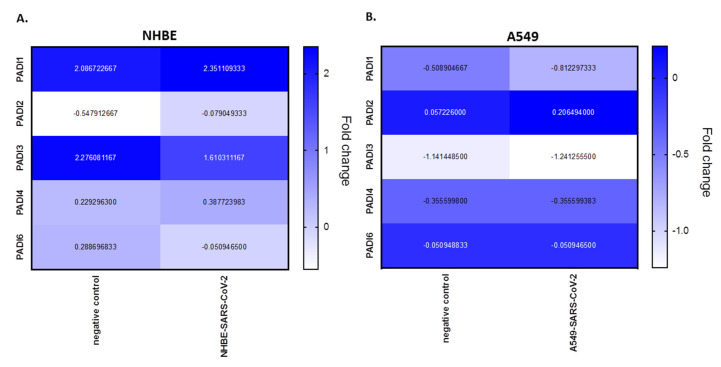
PRJNA615032 BioProject data was analysed for fold change expression for PADI mRNA levels in SARS-CoV-2-infected cell lines, compared with mock. (**A**) NHBE (human bronchial epithelial) cell lines; (**B**) A549 (adenocarcinoma human alveolar basal epithelial) cell lines. Heat map metric is given as blue bar between normalised mRNA levels; negative control represents the mock.

PADI mRNA expression levels were analysed from BioProject PRJNA631753, assessing human bronchial epithelial cell line (NHBE) and the adenocarcinoma human alveolar basal epithelial cell line (A549), infected with SARS-CoV-2, compared with mock infected cells. According to this analysis, in NHBE cells there is upregulation of PADI1 (1.14-fold), PADI2 (7.88-fold) and PADI4 (1.69-fold), in response to SARS-CoV-2 infection, but downregulation in PADI3 (−1.41-fold) and PADI6 (−5.65-fold) mRNA expression, compared with mocks. In the adenocarcinoma lung cells (A549), only PADI2 was upregulated (3.61-fold), while downregulation was observed for PADI1 (−1.60-fold) and PADI3 (−1.09-fold), but no effect was seen on PADI4 or PADI6 in SARS-CoV-2-infected, versus mock infected, cells (Figure 3).

### 2.3. Protein–Protein Interaction Network Identification of Human PAD Isoforms 1–6

**Figure 4:**
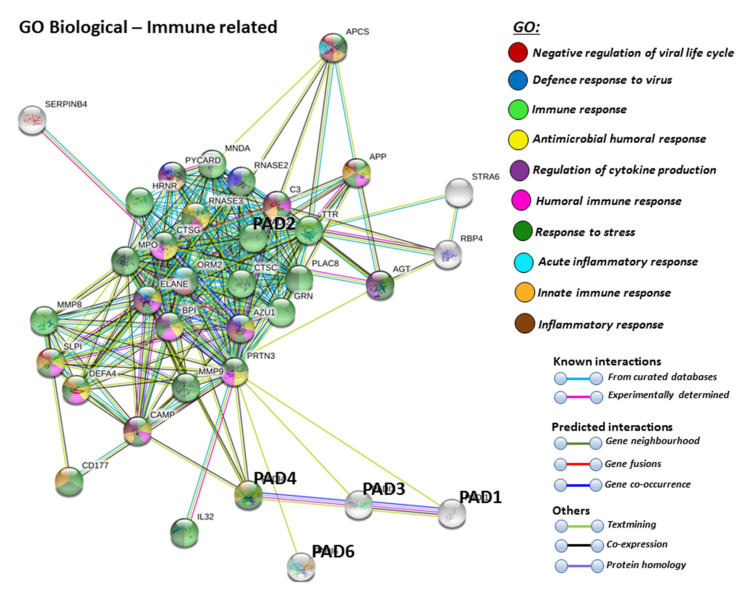
Biological GO (gene ontology) pathways for all human PAD isozymes, relating to immunity. Immune related GO biological pathways are highlighted for protein–protein interaction networks of binding partners with all PAD isozymes (PAD 1,2,3,4,6) as a group. Colour code for the different pathways is shown within the figure. Individual coloured lines represent specific protein interactions that have been identified through interactions that are known (referring to experimentally determined connections and curated databases), through interactions that are predicted (referring to gene fusion, gene neighbourhood, gene co-occurrence) or through interactions identified via text mining, co-expression or protein homology (please refer to the colour key provided in the figure for the different connective lines) (PPI enrichment *p*-value: < 1.0 × 10^−16^).

For the prediction of the protein–protein interaction networks of the PAD isoforms under study, the Human PAD protein IDs were submitted to Search Tool for the Retrieval of Interacting Genes/Proteins (STRING) analysis (https://string-db.org/), and analysed for Gene Ontology (GO) and Kyoto Encyclopaedia of Genes and Genomes (KEGG) pathways. When assessing GO biological pathways for all PADs as a group, significant protein–protein interaction networks were observed, with binding partners related to viral life cycle regulation, viral defence responses, and humoral, innate, acute and inflammatory immune responses, including antimicrobial immune responses, stress response and the regulation of cytokine production (Figure 4).

**Figure 5:**
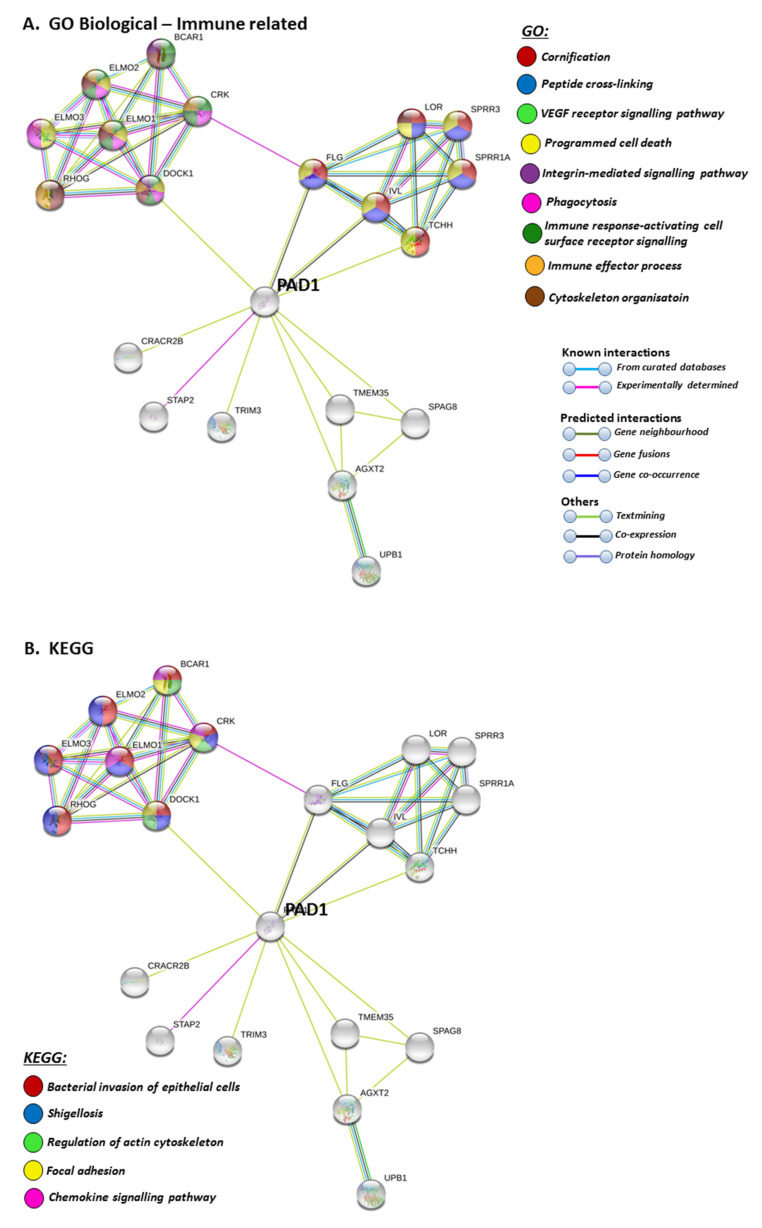

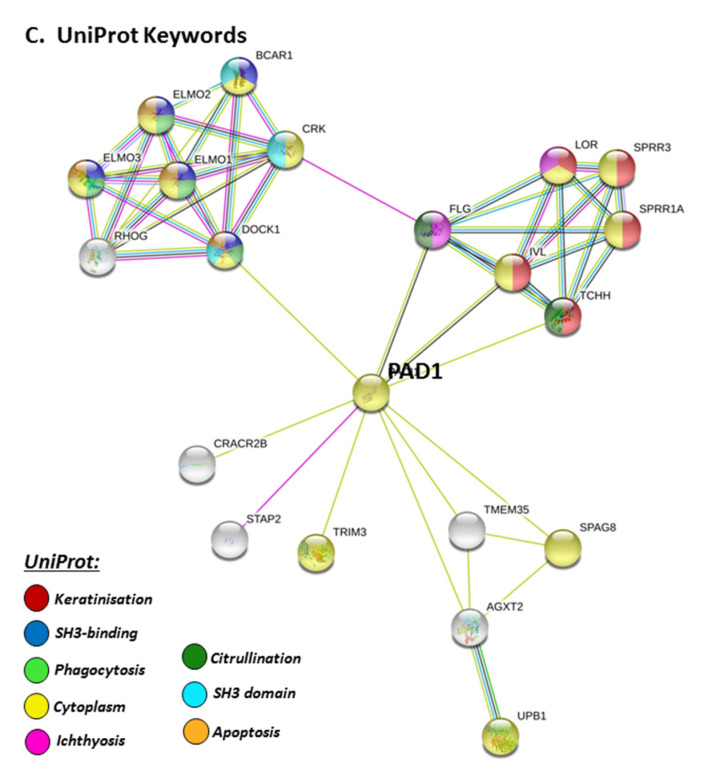
GO and KEGG analysis for PAD1, highlighting GO biological pathways (**A**) KEGG pathways (**B**) and UniProt keywords (**C**). Colour code for the different pathways is shown within the figure. Individual coloured lines represent specific protein interactions that have been identified through interactions that are known (referring to experimentally determined connections and curated databases), through interactions that are predicted (referring to gene fusion, gene neighbourhood, gene co-occurrence) or through interactions identified via text mining, co-expression or protein homology (please refer to the colour key provided in the figure for the different connective lines) (PPI enrichment *p*-value: 8.01 × 10^−8^).

STRING pathways for PAD1 revealed a range of immunological GO pathways, including phagocytosis, VEGF signalling, immune effector processing and programmed cell death (Figure 5A), while the KEGG pathway relates to infection and associated immune responses (Figure 5B). Roles in skin related responses, as well as immune responses, were further confirmed with UniProt keywords, which included skin diseases, phagocytosis, citrullination/deimination and apoptosis (Figure 5C).

**Figure 6:**
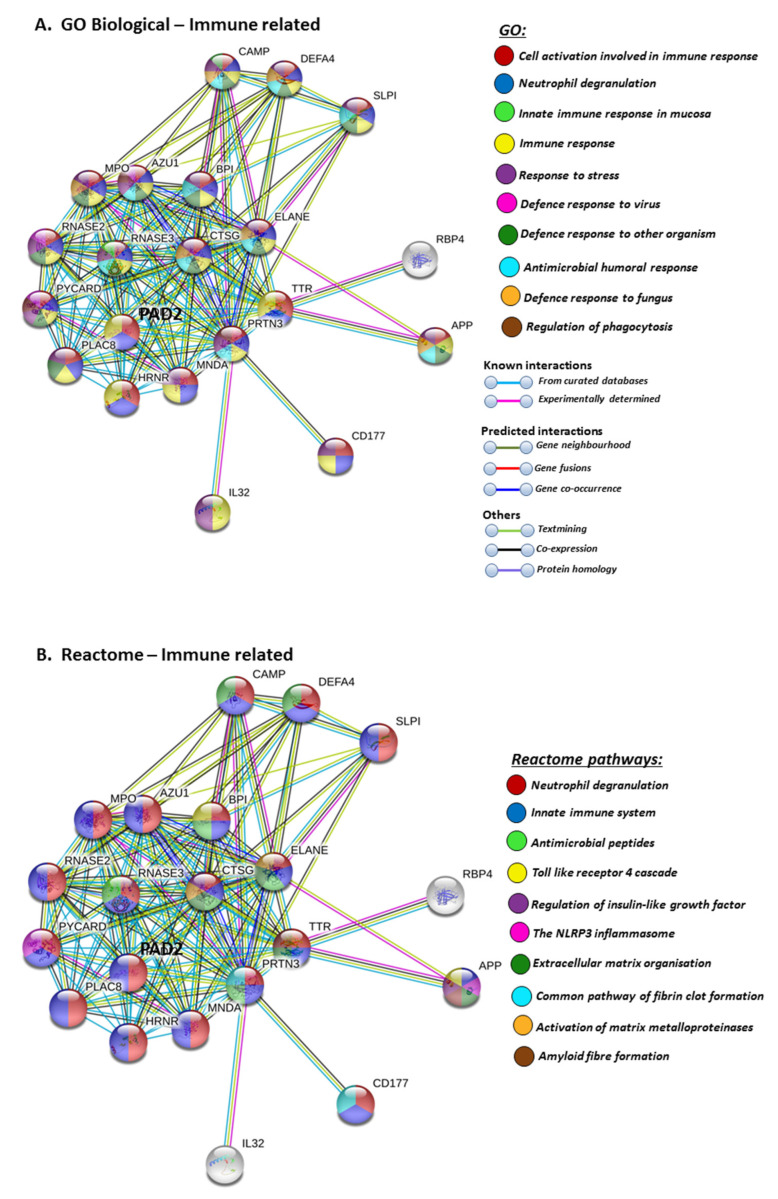

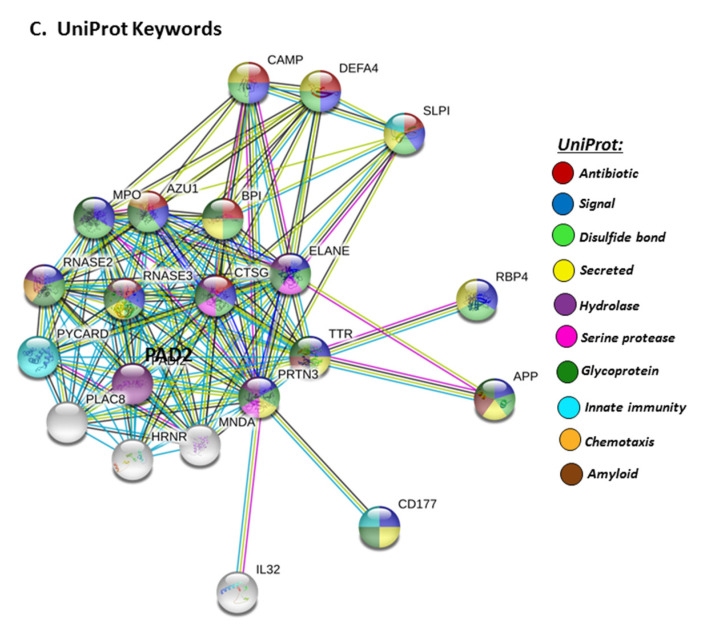
GO and Reactome pathway analysis for PAD2, highlighting immune related GO biological pathways (**A**) Immune related Reactome pathways (**B**) UniProt keywords (**C**) Colour code for the different pathways is shown within the figure. Individual coloured lines represent specific protein interactions that have been identified through interactions that are known (referring to experimentally determined connections and curated databases), through interactions that are predicted (referring to gene fusion, gene neighbourhood, gene co-occurrence) or through interactions identified via text mining, co-expression or protein homology (please refer to the colour key provided in the figure for the different connective lines) (PPI enrichment *p*-value: < 1.0 × 10^−16^).

Furthermore, PAD2 protein interaction networks indicate a strong role in immune responses, including innate immunity, mucosal immunity, response to stress, anti-microbial, antifungal responses and, in particular, defence responses to viruses. Regulation of phagocytosis and neutrophil degranulation are also linked to PAD2 and its protein interaction partners (Figure 6A). The reactome pathways for the PAD2 protein networks also verified a strong immune regulatory role (Figure 6B), and this correlated with identified UniProt keywords (Figure 6C).

**Figure 7:**
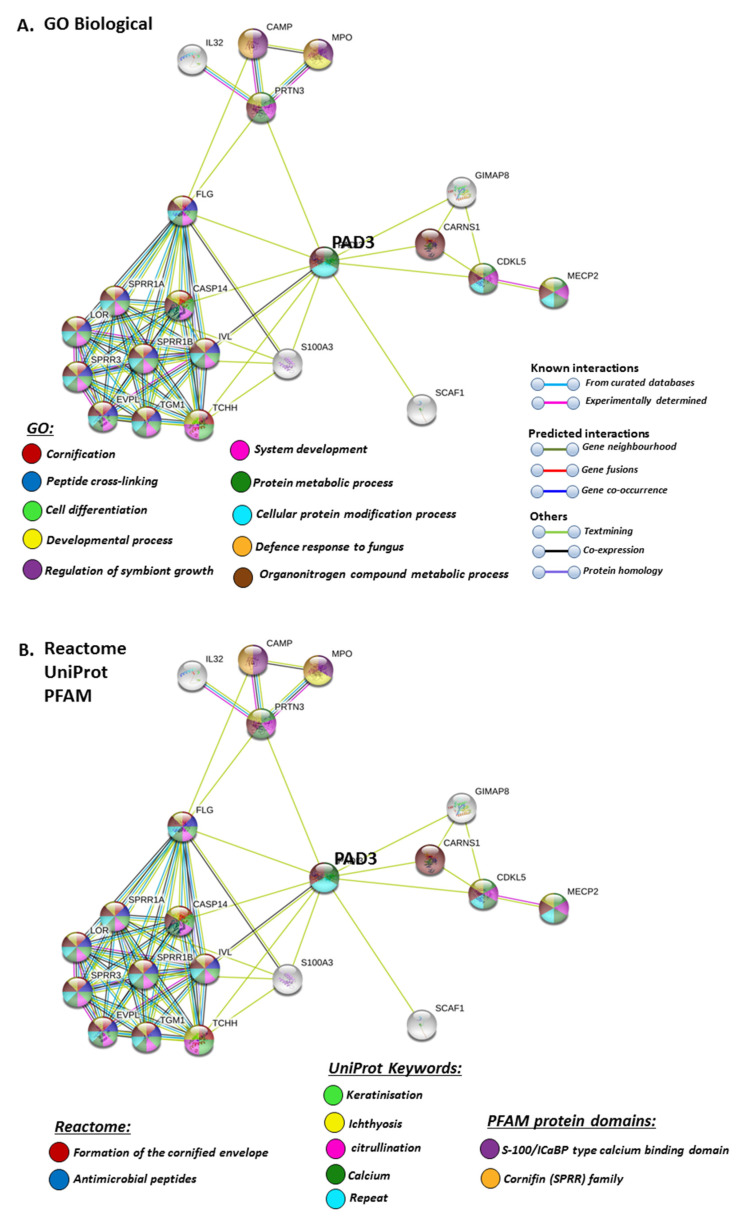
GO and Reactome pathway analysis for PAD3, highlighting GO biological pathways (**A**) Reactome pathways, UniProt keywords and PFAM protein domains (**B**) Colour code for the different pathways is shown within the figure. Individual coloured lines represent specific protein interactions that have been identified through interactions that are known (referring to experimentally determined connections and curated databases), through interactions that are predicted (referring to gene fusion, gene neighbourhood, gene co-occurrence) or through interactions identified via text mining, co-expression or protein homology (please refer to the colour key provided in the figure for the different connective lines) (PPI enrichment *p*-value: 2.22 × 10^−16^).

STRING analysis for PAD3 revealed biological GO pathways relating to skin physiology, cellular protein modification processes and cell differentiation, development, symbiosis and immune responses (Figure 7A), while Reactome pathways highlighted cornification and antimicrobial responses (Figure 7B). The Uniprot keywords related to skin-diseases and calcium-mediated pathways (Figure 7B), and this was also reflected in PFAM (protein families) protein domains highlighting pathways for calcium and cornification (Figure 7B).

**Figure 8:**
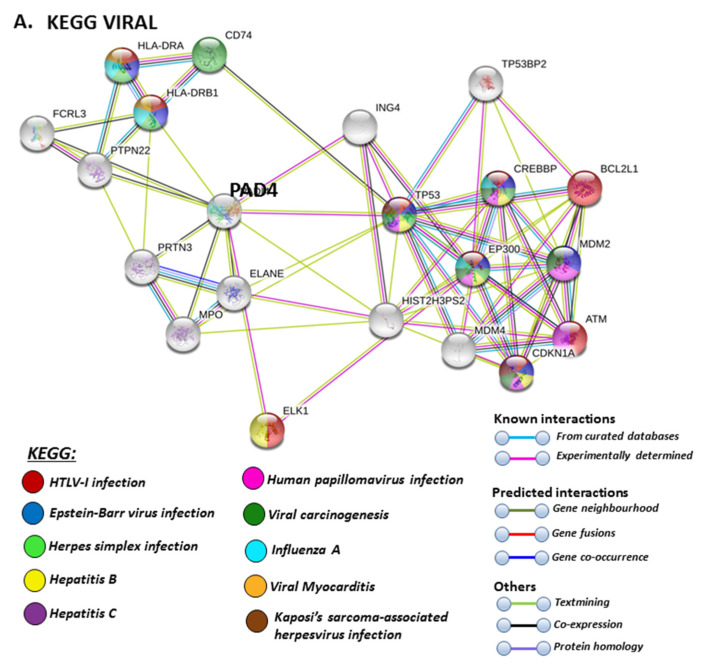

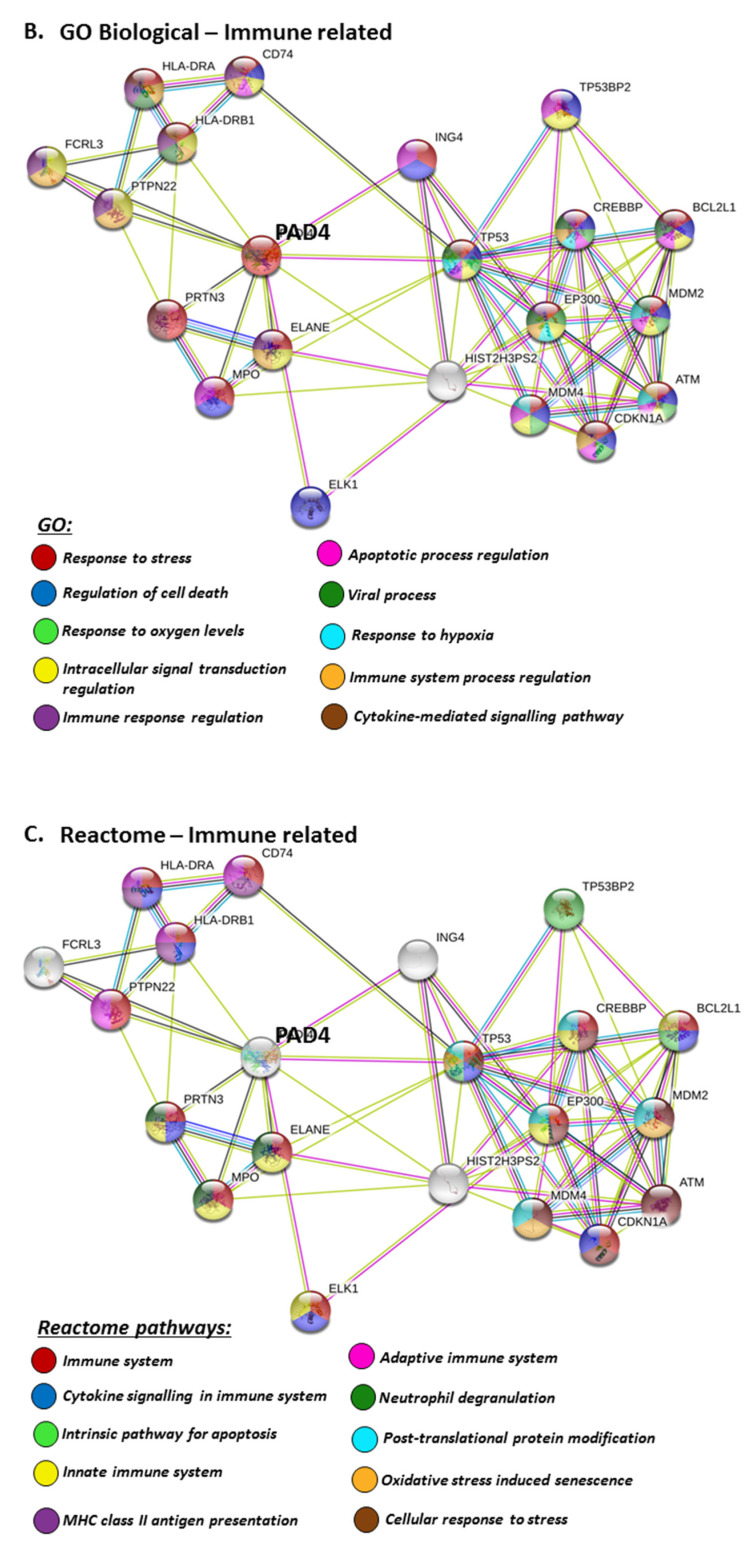

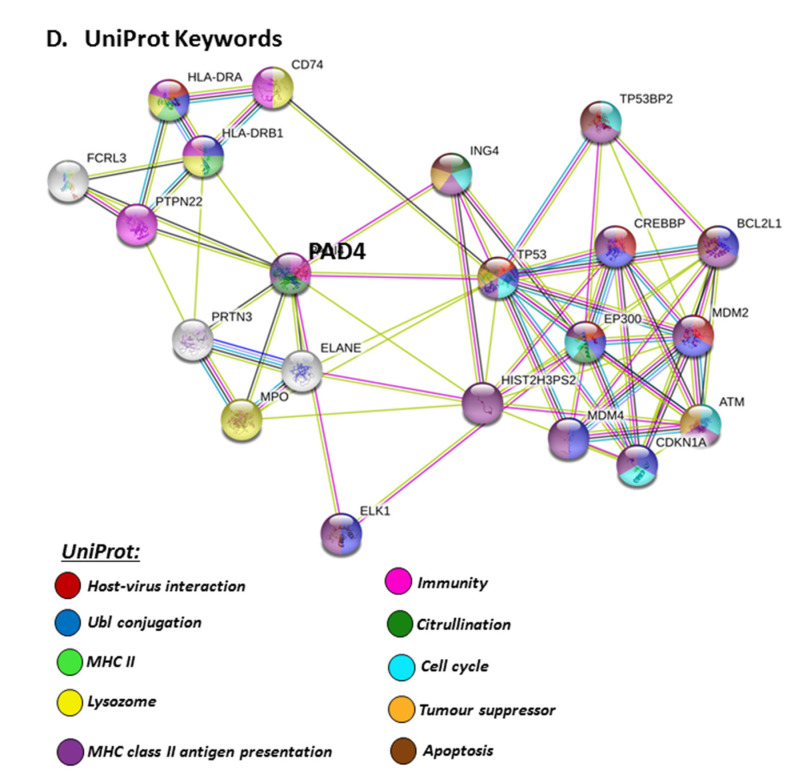
KEGG, GO and Reactome pathway analysis for PAD4, highlighting pathways relating to viral infections (**A**) Immune related GO biological pathways (**B**) Immune related Reactome pathways (**C**) and UniProt keywords (**D**) Colour code for the different pathways is shown within the figure. Individual coloured lines represent specific protein interactions that have been identified through interactions that are known (referring to experimentally determined connections and curated databases), through interactions that are predicted (referring to gene fusion, gene neighbourhood, gene co-occurrence) or through interactions identified via text mining, co-expression or protein homology (please refer to the colour key provided in the figure for the different connective lines) (PPI enrichment *p*-value: 1.21 × 10^−5^).

The PAD isozyme which was found to be most involved in viral responses via STRING analysis was PAD4, and the identified viral KEGG pathways for PAD4 via STRING are highlighted in Figure 8A. The viral pathways identified included Hepatitis C, HTLV-I, Epstein–Barr virus, Herpes simplex, Hepatitis B, papilloma virus, Influenza A, Kaposi’s sarcoma-associated herpes virus infection (KSHV), viral carcinogenesis and viral myocarditis (Figure 8A). The immune related GO biological pathways are summarised in Figure 8B, while immune related reactome pathways for PAD4 are highlighted in Figure 8C, and the identified UniProt keywords in Figure 8D.

**Figure 9:**
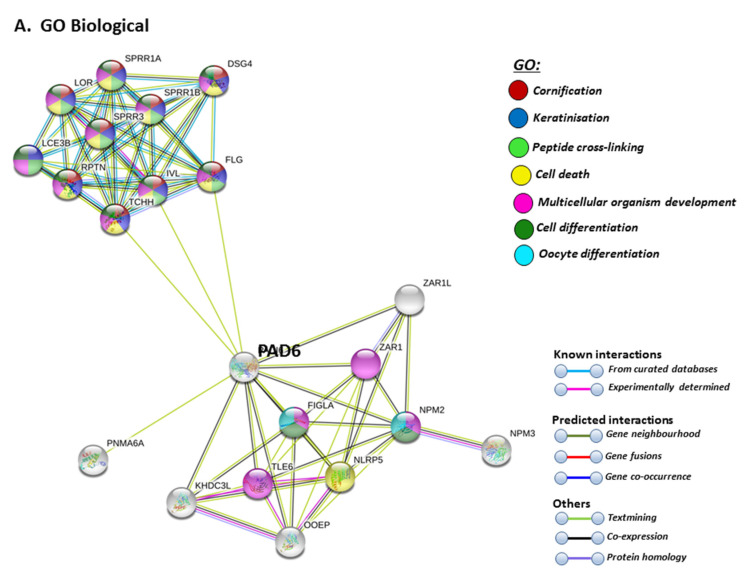

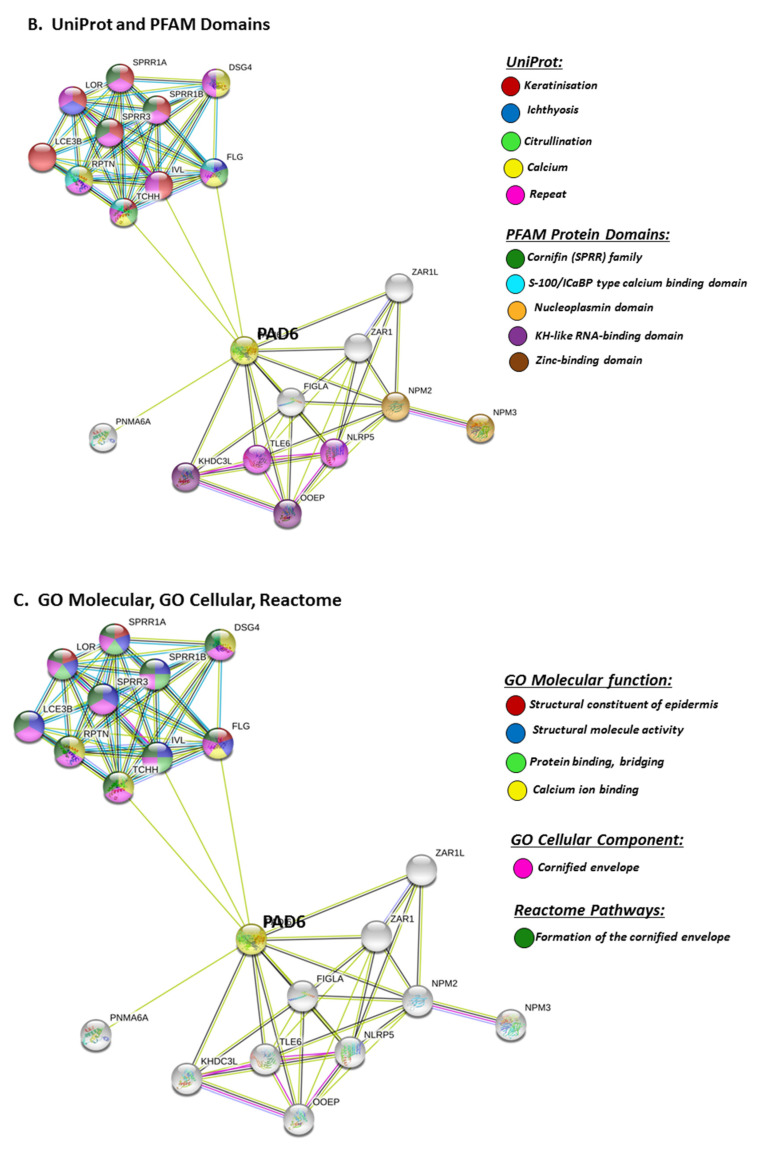
GO and KEGG analysis for PAD6, highlighting GO biological pathways (**A**) UniProt keywords and PFAM domains (**B**) and GO Molecular, GO cellular and Reactome pathways (**C**) Colour code for the different pathways is shown within the figure. Individual coloured lines represent specific protein interactions that have been identified through interactions that are known (referring to experimentally determined connections and curated databases), through interactions that are predicted (referring to gene fusion, gene neighbourhood, gene co-occurrence) or through interactions identified via text mining, co-expression or protein homology (please refer to the colour key provided in the figure for the different connective lines) (PPI enrichment *p*-value: 1. 0 × 10^−16^).

STRING analysis for PAD6 confirmed its known function in developmental and pre-implantation processes, but the GO pathways also highlight dual processes in skin physiology as well as immunity, involving cell differentiation and cell death (Figure 9A). UniProt keywords relate to calcium processes and skin physiology, while PFAM domains relate to similar functions, including calcium- and zinc-mediated processes (Figure 9B). GO pathways for molecular function highlight epidermal, structural and calcium binding pathways (Figure 9C), and cornification is further supported by GO Cellular component pathways and reactome pathways (Figure 9C). The link to keratinization/cornification and skin is unexpected, as PAD6 is not known to be expressed in the epidermis, and this link is only established via text mining (lime green line) linking PAD6 with Filaggrin (FLG), Involucrin (IVL) and Trichohyalin (TCHH) (Figure 9).

## 3. Discussion

PADs play a multiplicity of roles in inflammatory diseases, including chronic and infectious diseases. The roles for PADs in anti-viral responses have previously been identified, including via the generation of NETosis [29], which can be mediated by PAD4. Higher amounts of cyclic citrullinated/deiminated peptides in sera have indeed been related to infectious diseases, including a number of viral, bacterial and parasitic infections [75,76]. Deimination has also been identified to modulate chemokines in anti-HIV responses [30]. Furthermore, as PADs are phylogenetically conserved proteins, and are utilised by various pathogens for immune evasion, their roles in infection, as well as in zoonosis, may be of considerable interest. Co-infections or opportunistic infections with other pathogens may also be of importance in the interplay with viral infections, and PAD homologues in some bacteria have, for example, been shown to be able to act as an effective anti-viral agent [77]. The roles for PADs in virus host–pathogen interactions may also be of considerable interest in SARS-CoV-2 infection, as well as in secondary bacterial infections, and in relation to other multifaceted comorbidities observed in COVID-19.

Our present study has identified that PADI1, 2, 3 and 4 were most modulated in SARS-CoV-2-infected lung tissues (based on the analysis of samples from five patient cases), or lung-derived cells. Furthermore, PADI2 and PADI4 were the isozymes showing highest variation in SARS-CoV-2-infected lung tissue, with PADI4 showing more dominance. PAD4 is indeed linked to multiple viral KEGG pathways, and is furthermore considered one key-driver of NETosis [29]. There was some variation in other PAD isozymes regarding whether they were elevated or reduced, and this may be somewhat reflected in the high individual variation observed for all PADI isozymes in the five cases present in the BioProject data. Elevated PADI4 may lead to more active defences and an increased NETosis, but may possibly also result in over-activation of inflammatory responses and destruction of surrounding tissue [78,79]. On the other hand, reduced PADI4 levels may contribute to less active defences against viral infection, due to gene regulatory changes, changes in deimination of immune related proteins, or impaired NETosis in these individuals. It remains to be further investigated whether the virus may manipulate PADI4 expression, as an immune evasion mechanism, or for example whether individuals with lower PADI4 expression are more prone to SARS-CoV-2 infection.

PADs are furthermore implicated in other COVID-related comorbidities, such as myocardial responses [5], and myocardial deimination/citrullination has been observed in coxsackievirus B3-induced viral myocarditis [80]. Viral myocarditis has furthermore been related to KEGG pathways identified for deiminated proteins in comparative bovine animal models [48]. The involvement of modulated PADI expression in viral-induced myocarditis, including in relation to COVID-19, may therefore be of considerable interest. Myocardial deimination has also been assessed in relation to rheumatoid arthritis (RA) [81], one of the major PAD-associated diseases [82], and interestingly, anti-RA drugs have been considered candidate therapies in COVID-19 [83,84]. PADI3 was here the overall isozyme which was observed to be elevated in heart tissue in SARS-CoV-2 biopsies, while PADI4 was elevated in the heart tissue of one case. These findings will need further validation in larger cohorts, and also with the use of tissue-specific controls.

The individual variation of PADI isozyme expression observed here in SARS-CoV-2-infected patient biopsies is of considerable interest, as it aligns with the great individual variation and the broad spectrum of immunological response in COVID-19 patients, ranging from non-existent to cytokine storm, as well as other comorbidities such as myocarditis, stroke and extreme vascular responses. Furthermore, as the different PADIs, based on BioProject analysis, do respond differently in different patients, their deiminated target proteins also need further evaluation in order for us to build a larger picture of downstream effects. Using a range of comparative animal models, we have previously identified that target proteins of deimination link to a range of immune and pathogenic pathways, including anti-viral ones [47,48]. Furthermore, PADs are linked to a range of autoimmune diseases, some of which are comorbidities with COVID-19. Therefore, it will be crucial to evaluate downstream targets, namely post-translationally deiminated proteins, as these will be modified in structure and function due to PAD activation and dysregulation, possibly also influenced by PADI RNA levels per se, although in addition the Ca^2+^-mediated activation of the PAD enzymes is crucial for the post-translational deimination of target proteins. One major shortcoming in the BioProject study is that the control samples are from the lung only, and therefore PADI-expression levels in the other SARS-CoV-2 tissues, besides lung, cannot be directly compared with the corresponding tissue-specific control.

The role for PADs in secondary bacterial and/or other opportunistic infections is also of considerable importance. PADs are associated with a range of bacterial infections, including their role in endotoxic shock, also relating to pulmonary dysfunction [32,36] and sepsis [33,34], where PADI4 levels have been associated with ICU (intensive care unit) mortality [35], as well as in antibiotic resistance, where roles for PAD2 and PAD4 have been described [39]. Deimination of anti-microbial peptides during rhinovirus infection has previously been identified [31], and furthermore, deimination of chemokines has been shown to modulate immune responses against HIV [30], and may be a downstream factor in PADI modulation in COVID-19, warranting further exploration. In addition, the generation of NETosis has been related to airway obstruction during respiratory syncytial virus disease [40], and as this is partly PAD4-related, such effects in response to SARS-CoV-2 will require further assessment.

In the BioProject in vitro models, two different lung cell lines were assessed following SARS-CoV-2 infection, namely human bronchial epithelial cells (NHBE) and an adenocarcinoma human alveolar basal epithelial cell line (A549). From this data, compared with control mock-infected cells, we found that PADI1, PADI2 and PADI4 mRNAs were elevated in the infected NHBE cells (but PAD3 and PADI6 were downregulated), while only PADI2 was elevated in infected A549 cells (but PADI1 and PADI3 were downregulated). This indicates differences between normal versus cancerous lung cells, indicating a link between PADI1, PADI2 and PADI4 in normal lung tissue responses and SARS-CoV-2 infection, which aligns with the reported roles of PADI1, 2, and 4 isoforms in immune responses, as well as for PADI2 and PADI4 in anti-viral responses specifically. In the adenocarcinoma lung cells, PADI4 mRNA levels stayed similar in SARS-CoV-2-infected compared to mock infected cells, possibly indicating lower viral defence mechanisms via PAD4, while PADI2 was elevated, and may be the dominating PADI isoform acting as part of its anti-viral and pathogenic immune related functions in this cell line. PAD4 has previously been shown to be elevated in lung adenocarcinoma, to play roles in A549 EMT transition [85,86] and to be important for PAD4-mediated NETosis in lung epithelial malignancies [87], while PADI2 has been related to prostate cancer proliferation [88] and is elevated in breast cancer [89]. Therefore, it may be speculated that the increased PADI2 expression levels in A549 cells may lead to alterations in these cancer cells in order to promote cell survival and pathogenesis. It must be stressed, though, that the current study only evaluated PADI mRNA levels, but not protein levels, as only mRNA data are available in the BioProjects.

The roles of PADs in mucosal immunity are important. PADI expression has been described in mammalian mucosal tissues, including uterus, gastric and colon tissues [90,91], where changes in deimination are associated with ulcerative colitis and cancer pathogenesis [92], and in bronchial and alveolar mucosa in response to harmful stimuli [50], as well as in lung inflammation and cystic fibrosis [51,52,53]. Indeed, in comparative animal models, we have recognised important roles for PADs and PAD-mediated NETosis in teleost mucosa, which mirrors the human mucosal surfaces present in the respiratory tract, intestine and uterus [41,43]. The ability of viruses to induce NETosis is indeed recognised [93], and NETosis is also associated with inflammatory responses and infection in various mucosal surfaces, including the gut [41,94], and in antimicrobial defence in the oral mucosa [95]. In the current study, we did find that PADI1, PADI2, PADI3 and PADI4 mRNA was low in SARS-CoV-2-infected bowel tissue compared with normal lung tissue, while PAD6 mRNA was not affected, and in jejunum PAD3 mRNA was low, compared with normal lung tissue. These findings will need to be validated against tissue-specific controls in future studies, and furthermore, although the mRNA levels are not elevated, activation of the PAD enzymes and the consequential downstream deimination in response to viral infection may still occur.

Another aspect that will need to be considered in future studies is the regulatory roles of PADs in extracellular vesicle (EV) release [39,54,55,56], which we have shown to be differently regulated by the different PAD isozymes [23]. EVs play major roles in infectious disease [57] and can also aid viral immune evasion [96]. The diverse roles for EVs in mucosal tissues, and their relevance in various mucosal-related diseases and in the physiological function of mucosal surfaces, are gaining attention [44]. Important roles for EVs have been implicated in oral mucosa and wound healing [97], intestinal inflammation and repair [98], host–pathogen interactions in intestinal infections [99], including via PAD-mediated pathways [69], and in intestinal mucosal immunity [100], as well as in airway tissue, lung disease and in allergic response [101,102,103,104]. Indeed, the regulation of EVs, their use as biomarkers and their application in the therapeutic intervention of COVID-19, have all recently been highlighted [61,62,63,105,106,107]. Therefore, the contribution of the different PAD isozymes in the regulation of EV release, also relating to tissue-specific PAD expression, will need to be explored in relation to SARS-CoV-2 infection in future in-depth studies.

Detailed exploration into the target proteins of PAD-mediated deimination in COVID-19 will need further assessment, as the subcellular distribution of deiminated proteins is tissue type-dependent [108]. The citrullinome of chronic diseases has been reported, including rheumatoid arthritis (RA), multiple sclerosis (MS) and Alzheimer’s disease (AD) [82,109,110], as well as in a range of taxa throughout the phylogenetic tree, both in plasma and the EV secretome [39,44,45,46,47,48,66,67,111]. Interestingly, the enrichment of deiminated proteins for KEGG pathways relating to viral infection has recently been identified in the serum and serum EVs of alligator and cow, both which are animals with unusual anti-viral responses [47,48]. Furthermore, both shark and llama nanobodies have recently been identified as targets of PADs, and as being post-translationally deiminated [45,46], as have cow immunoglobulins (Ig’s) [48], and this may be of considerable importance as these are currently being screened for their neutralising activity against SARS-CoV-2, with great promise for llama nanobodies [112,113]. The assessment of PAD-mediated pathways across taxa is therefore a valuable approach to furthering understanding of this phylogenetically conserved pathway, which still requires much exploration with respect to infectious, including zoonotic, diseases.

A somewhat unsuspected comorbidity of COVID-19 is stroke [12]. PADs and deimination have indeed been recognised as a significant component in hypoxic ischaemic brain injury [25,26], as well as in traumatic and blast brain injury [114,115], and may contribute to autoimmune dysfunction in the chronic pathology following such events. The relationship between SARS-CoV-2 infection and stroke may be somewhat linked with the high vascular inflammatory reaction and vascular component seen in many COVID-19 cases, including also effects on pericytes [4], and therefore also with putative effects on the blood–brain barrier. Interestingly, a recent study by our group identified that in pre-motor Parkinson’s disease (PD), the brain vasculature is heavily deiminated, identifying a novel contribution of PAD-mediated deimination to brain endothelial cell responses [116], possibly also occurring as a systemic inflammatory response. Deimination changes in the brain vasculature could possibly have implications with respect to the chronic CNS changes that have been suggested to possibly occur following SARS-CoV-2 infection, including in relation to age-related neurodegenerative disorders [117]. In the pre-motor PD brain vasculature, PAD4 was found to be the dominating PAD isozyme to be upregulated [116], and this isozyme also has the strongest link to viral infections, including SARS-CoV-2, as observed here in our current study. As COVID-19 is increasingly being acknowledged to have a heavy vascular component, indicating that endothelial cells may be essential contributors to the initiation and propagation of severe COVID-19 [4], deimination in the vasculature in multiple organs in response to SARS-CoV-2 infection remains to be further investigated. This may offer an explanation for the unusual vascular responses observed, as well as some of the unexplained stroke and other neurological aspects of COVID-19, which are possibly partly linked to deimination in the brain vasculature and the downstream effects, as previously discussed in relation to pre-motor PD (which also has a considerable inflammatory component) [116]. A recent link between COVID-19 and Guillain–Barré syndrome (GBS), an acute immune-mediated polyradiculoneuropathy, has also been revealed [118,119]. Although PADs are implicated in peripheral nerve diseases, a direct link to GBS has before now not been successfully established [120,121], although it has to be highlighted that case studies have mainly assessed the presence of citrullinated auto-antibodies [122], and therefore other PAD-mediated pathways remain to be investigated in GBS. Furthermore, a hypoxic component, due to the severe lung reaction, may also result in downstream activation of the PAD-mediated pathways that have been shown to contribute to hypoxic injury, including in the CNS [25,26,123]. In addition, the roles of PADs in inflammatory diseases are well acknowledged, including via PAD4-mediated NETosis [124], which has also been observed in the impaired CNS [24,25,116]. Indeed, the accumulation and extravasation of neutrophils is one of the responses of endothelial cells to SARS-CoV-2 infection, and it is suggested to contribute to tissue damage [4], possibly also inducing PAD-mediated NETosis. Importantly, as our paper goes to press, a surveillance study has just been published in the Lancet highlighting neurological complications in COVID-19, calling out for identification of, and investigations into, putative underlying mechanisms [125].

The effects on the kidneys of COVID-19 have been frequently observed, with clinical presentation ranging from mild proteinuria to progressive acute kidney injury [9,10]. In previous studies, PAD inhibition has been shown to protect against kidney, skin and vascular disease in a mouse model of lupus, including via the disruption of NET formation [126]. PAD4 has also been found, in patients, to be involved in the pathogenesis of anti-neutrophil cytoplasmic antibody (ANCA)-associated vasculitis (AAV) [127]. Furthermore, neutrophil-derived PAD4 has been linked to ischaemia–reperfusion kidney injury [128], where PAD4 has been found to promote renal tubular inflammation, neutrophil infiltration, and NF-κB activation [129]. In our current study, we did observe some changes in PAD expression in SARS-CoV-2-infected kidney biopsies, including elevation of PADI4, PADI1, PADI3 and PADI6 mRNA in some of the five cases. Interestingly, both PAD1 and PAD3 are strongly related to skin diseases and physiology [74] and also have implications in immunity, PAD3 has for example been identified in neuroinflammation [24]; however, they have not been studied in relation to kidney injury, which hitherto has mainly focussed on PAD4. The roles of other PADs, besides PAD4, therefore warrants further exploration in relation to kidney injury in COVID-19.

Our current study identified that in the liver, PADI2 and PADI4 were elevated in SARS-CoV-2 biopsies, compared with control lung biopsies, and the roles of PAD2 have previously been identified in liver fibrosis [130], while PAD4-mediated NETosis has been linked to liver vasculature and liver injury in bacterial infection [131]. In fat, PADI2 mRNA was downregulated, while the other PADIs were unchanged, compared with normal control lung tissue. In marrow, PADI2 and PADI3 were elevated in some SARS-CoV-2 biopsies, and interestingly PADI2 was found to be elevated in marrow mesenchymal stem cells, which upregulates IL-6 via histone H3 deimination in multiple myeloma, contributing to chemo-resistance [132]. Interestingly, PADI1 was elevated in the skin of SARS-CoV-2-infected biopsies, and is known for its roles in skin physiology and diseases [74]. The high levels of PADI1 observed here in the SARS-CoV-2 skin biopsies may possibly contribute to some of the cutaneous manifestations related to COVID-19, which include an erythematous rash, urticarial, chickenpox-like vesicles, acral lesions (“COVID toes”) and livedoid lesions [11,133].

In summary, our findings suggest roles for PADs in SARS-CoV-2 infection, based on data extracted from the public data on BioProjects for autopsy and in vitro samples. Our reported findings will need further validation in larger patient cohorts, including tissue-specific controls for all tissue types, as the current autopsy samples only used lung tissue as control. The identification of downstream deiminated target proteins also remains a topic of future in-depth investigation. Overall, PADI isozyme mRNA expression was found to show high individual variability, while PADI4 was a dominant isoform in relation to SARS-CoV-2 infection overall. Importantly, as PADs are phylogenetically conserved and take on multiple roles in host–pathogen interactions throughout the phylogenetic tree, their role in zoonotic diseases is of great interest, and remains a field of further study. Our findings are promising for the assessment of PAD-isozyme specific inhibitors, which have been developed and validated in a range of chronic and CNS disease models, for possible use following SARS-CoV-2 infection.

## 4. Materials and Methods

### 4.1. In Silico Analysis of BioProject Data for PADI Isozyme mRNA Expression in SARS-CoV-2 Versus Control Lung Tissues

BioProject data was obtained from PRJNA615032 BioProject trancriptome data [134], which includes lung biopsies from SARS-CoV-2-infected patients and healthy volunteers, as well as mock and SARS-CoV-2-infected primary normal human bronchial epithelial cells (NHBE) and lung cancer (A549) cell lines. Additionally, BioProject public data from PRJNA631753 was also utilised, where biopsies from multiple tissues from 5 patients were assessed in comparison to control lung tissue. The data have been deposited with links to BioProject accession number PRJNA615032 and PRJNA631753 (Ting Lab, Cancer Center, Massachusetts General Hospital, https://www.ncbi.nlm.nih.gov/bioproject/?term=PRJNA631753) in the NCBI BioProject database (https://www.ncbi.nlm.nih.gov/bioproject/).

In the present study, all the selected data was reanalysed using the Rosalind bioinformatics server. Data analysis was performed according to 1.25-fold change between mock and infected cell lines (NHBE, human bronchial epithelial cells, and A549, adenocarcinoma human alveolar basal epithelial cells) in a data pool calculation for both cell lines at *p* < 0.05 significance level. Data was analysed using Rosalind (https://rosalind.onramp.bio/), with a HyperScale architecture developed by OnRamp BioInformatics, Inc. (San Diego, CA, USA). The row factor for NHBE mock vs. SARS-CoV-2-infected cells was *p* < 0.001, according to the heatmap plot presented. Other plot presentations present normalised data, which is filtered according to the Rosalind algorithm.

Trimming of reads was performed using Cutadapt [135]. Assessment of quality scores was performed using FastQC [136]. The resulting read alignment was performed with the *Homo sapiens* genome build hg19 for PRJNA631753 and with GRCh38 for PRJNA615032, where STAR [137] was used. Quantification of individual sample reads was carried out using HTseq [138], followed by normalisation using Relative Log Expression (RLE) and DESeq2 R library [139]. The read distribution graphs, percentages, identity heatmaps, as well as sample MDS plots, were generated using RSeQC, as part of the QC step [140]. Fold changes were calculated using DEseq2, which was also used to perform optional covariate correction and calculate *p*-values. Gene clustering for generation of the final heatmaps was performed using the Partitioning Around Medoids (PAM) method to show differentially expressed genes, using the fpc R library (https://cran.r-project.org/web/packages/fpc/index.html).

### 4.2. STRING Protein–Protein Interaction Network Analysis for PAD Isozymes

To predict and identify putative protein–protein interaction networks for the human PAD isoforms (PAD1,2,3,4 and 6), STRING analysis (Search Tool for the Retrieval of Interacting Genes/Proteins; https://string-db.org/) was carried out. The different protein networks were generated based on IDs of the PAD isozymes. The following parameters were applied in STRING: function selected was “search protein by name”, the chosen species database was “*Homo sapiens*”. Network analysis was further carried out by applying “basic settings” and “medium confidence”. Nodes are connected by differently coloured connecting lines, which represent interactions for the network edges, based on evidence as follows: “known interactions”, which are based on experimentally determined interactions or curated databases; and “predicted interactions”, which are based on co-expression, protein homology, gene fusion, gene co-occurrence, gene neighbourhood, or are established by text mining.

### 4.3. Statistical Analysis

BioProject transcriptomics data was analysed using Rosalind (https://rosalind.onramp.bio/), with a HyperScale architecture developed by OnRamp BioInformatics, Inc. (San Diego, CA, USA). Graphs and heatmaps were prepared using GraphPad Prism version 7.0 (GraphPad Software, San Diego, CA, USA). STRING analysis (https://string-db.org/) was used for prediction of protein-protein interaction networks. Significance levels were was considered as *p* ≤ 0.05.

## 5. Conclusions

The roles for the five different human PADI isozymes, in response to SARS-CoV-2 infection, are here analysed for the first time, based on transcriptome BioProject data from patients’ biopsies and in vitro experiments. While PADI4 seems particularly involved in SARS-CoV-2 infection, followed by PADI2, the other PADI isozymes may also play some roles, and in the five patients assessed, high individual variability was observed for all PADI isozymes, including PADI1, 3 and 6. It will therefore be necessary to evaluate PADI isozyme expression, alongside protein levels, in larger patient cohorts in further studies. The assessment of PAD-mediated effects on EV-regulation, and of deiminated proteins produced by PAD isozyme activation in the different tissues, is furthermore of pivotal importance, and the aim of future studies. Such analysis will allow for the identification of deiminated target proteins and disease-specific EV-signatures, and will increase current understanding of disease pathways relating to the wide range of symptoms and comorbidities observed in COVID-19. Our study highlights roles for PADs in SARS-CoV-2 infection, and identifies them as putative drug targets, including via PAD isozyme-specific targeting, for treatment in COVID-19.

## Supplementary Materials

The following are available online at https://www.mdpi.com/1422-0067/21/13/4662/s1, Figure S1: PAD1 expression in control lung biopsies and COVID-19 autopsies, Figure S2: PAD2 expression in control lung biopsies and COVID-19 autopsies, Figure S3: PAD3 expression in control lung biopsies and COVID-19 autopsies, Figure S4: PAD4 expression in control lung biopsies and COVID-19 autopsies, Figure S5: PAD6 expression in control lung biopsies and COVID-19 autopsies.

## Abbreviations

AAV	AAV (antineutrophil cytoplasmic antibody (ANCA))-associated vasculitis
AD CNS	Alzheimer’s disease Central nervous system
CoV	Coronavirus
COVID-19	Coronavirus disease 2019
ETM	Epithelial-mesenchymal transition
EVs	Extracellular vesicles
GBS	Guillain-Barre syndrome
Ig	Immunoglobulin
KEGG	Kyoto encyclopedia of genes and genomes
NETosis	Neutrophil extracellular trap formation
PAD	Peptidylarginine deiminase
PD	Parkinson’s disease
RA	Rheumatoid arthritis
SARS	Severe acute respiratory syndrome
