# Supplementary figures and images for: Putative Roles for Peptidylarginine Deiminases in COVID-19
Source: Int J Mol Sci. 2020 Jun 30;21(13):4662. doi: 10.3390/ijms21134662 (PMC7370447; doi:10.3390/ijms21134662)

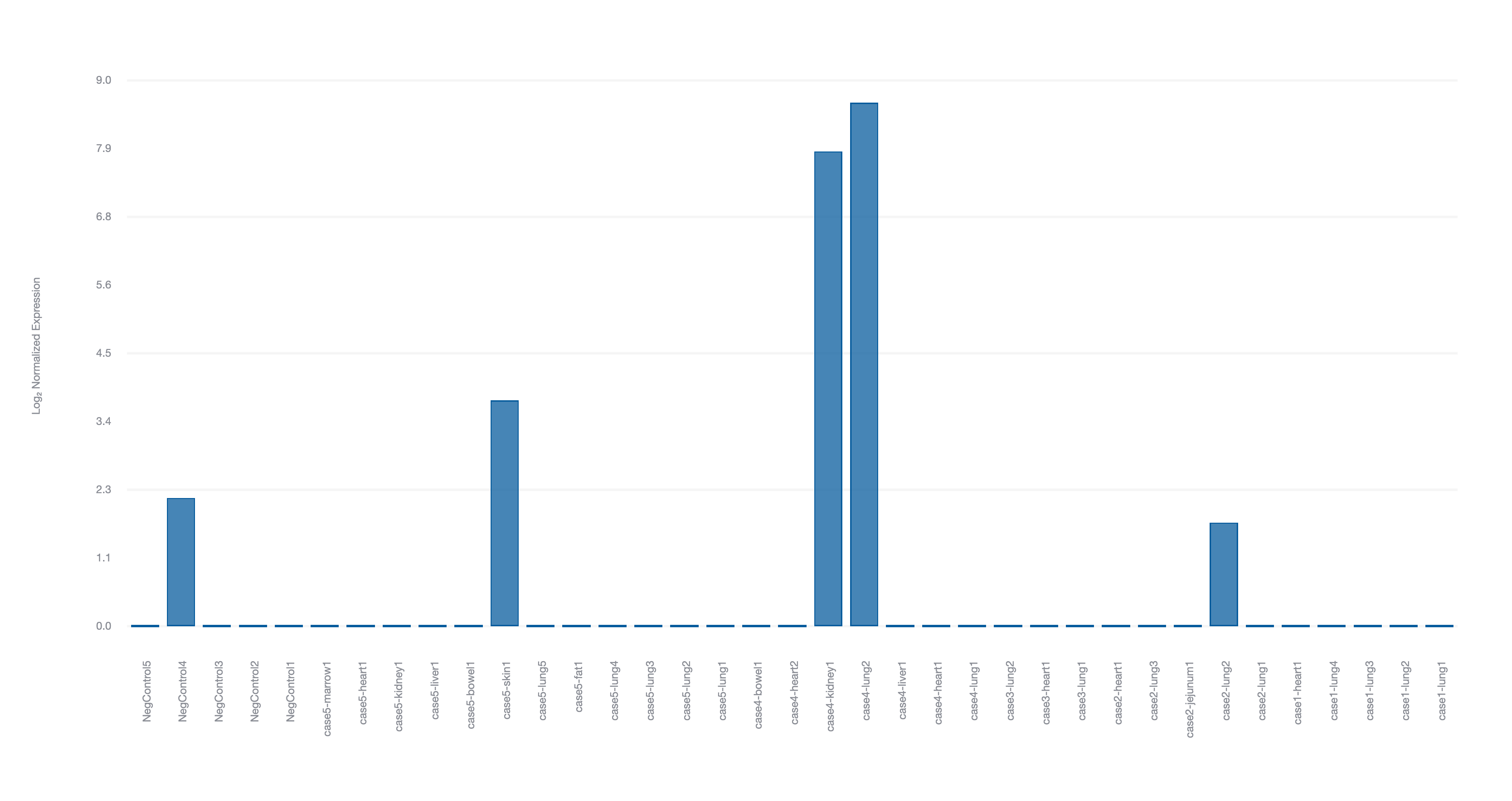

Supplement: Supplementary file 1 [file ijms-21-04662-s001.zip › Supp Fig S1 pad1 all tissues.png]

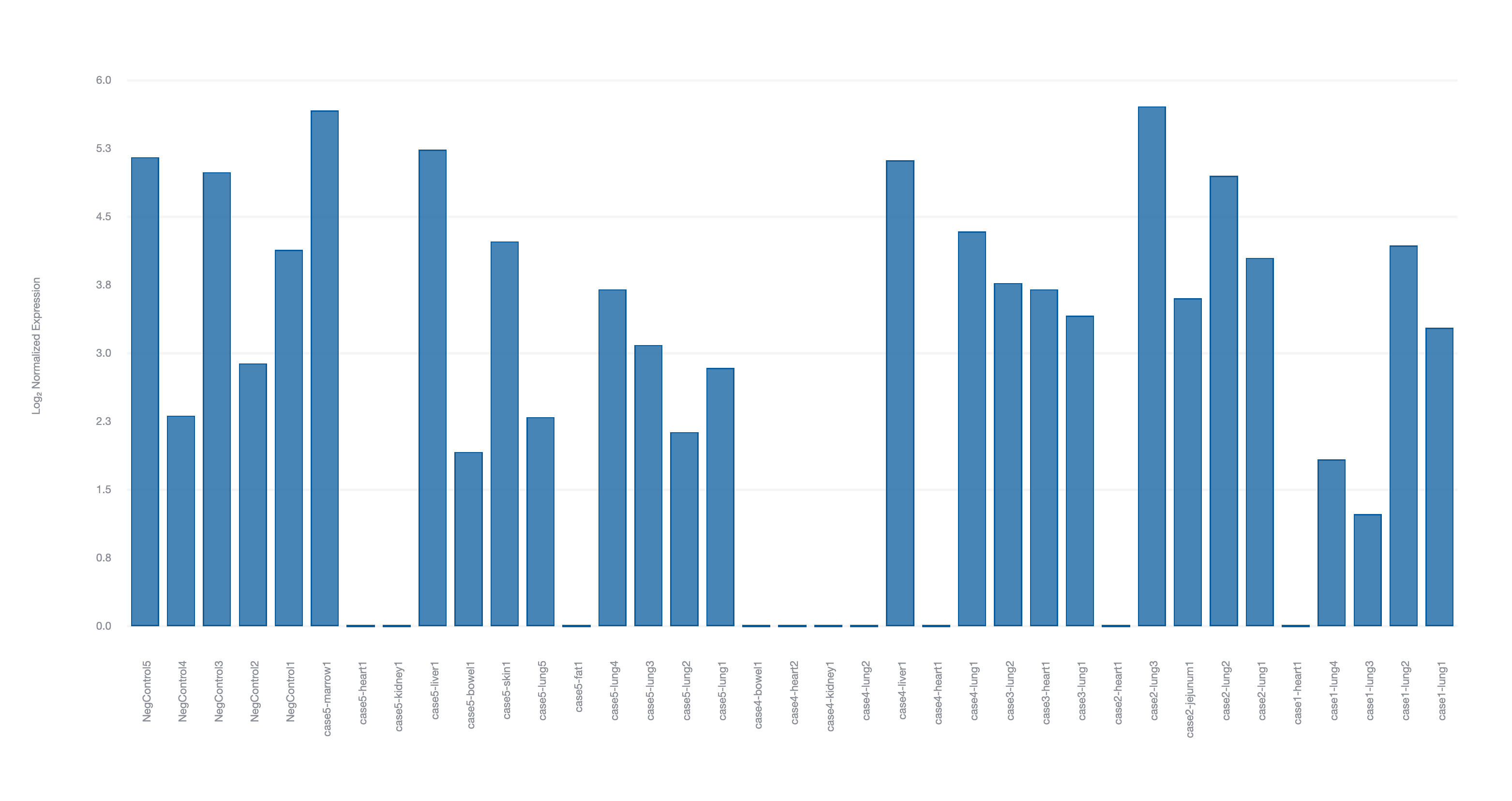

Supplement: Supplementary file 1 [file ijms-21-04662-s001.zip › Supp Fig S2 pad2 all tissues.png]

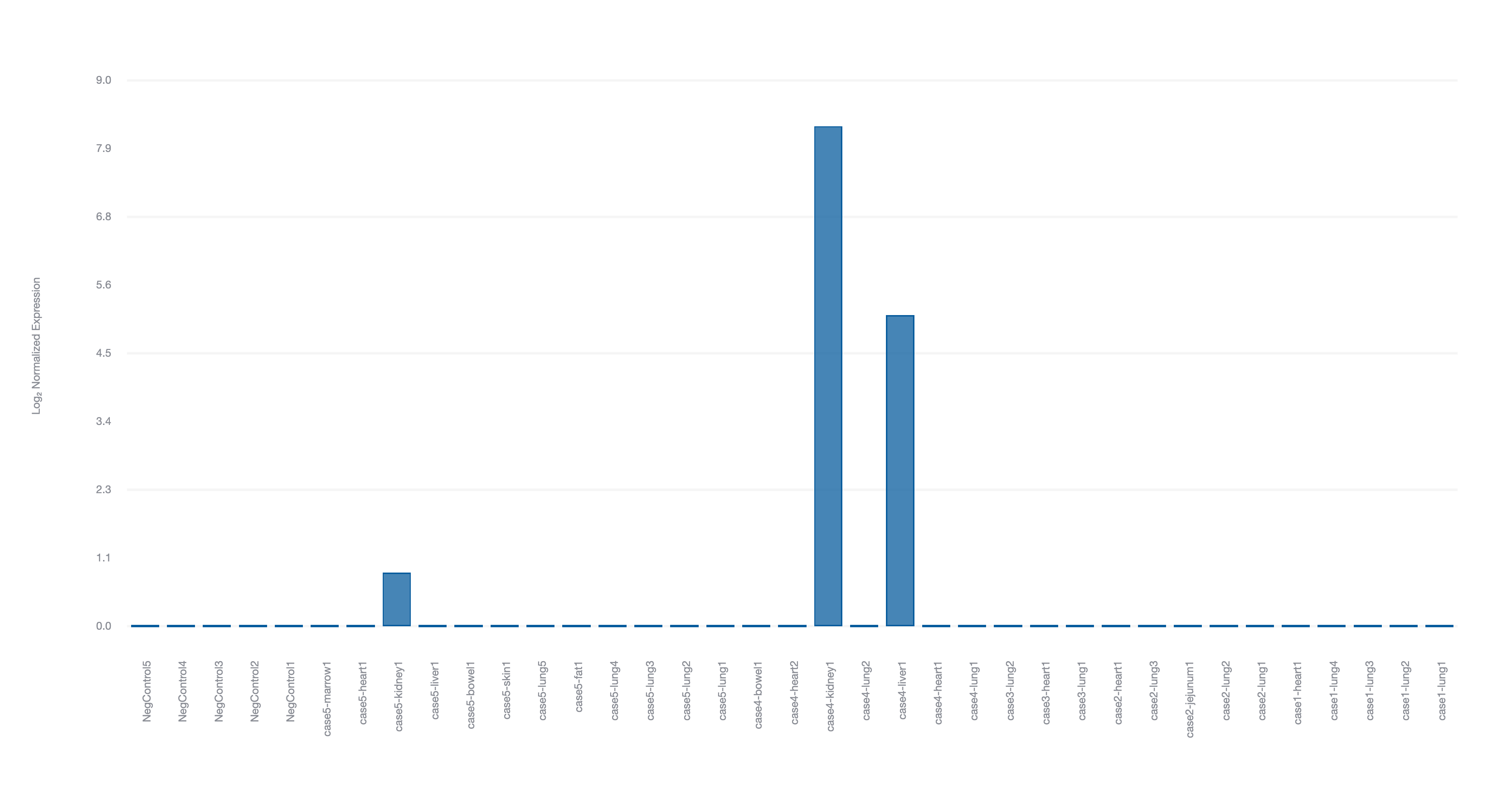

Supplement: Supplementary file 1 [file ijms-21-04662-s001.zip › Supp Fig S3 pad3 all tissues.png]

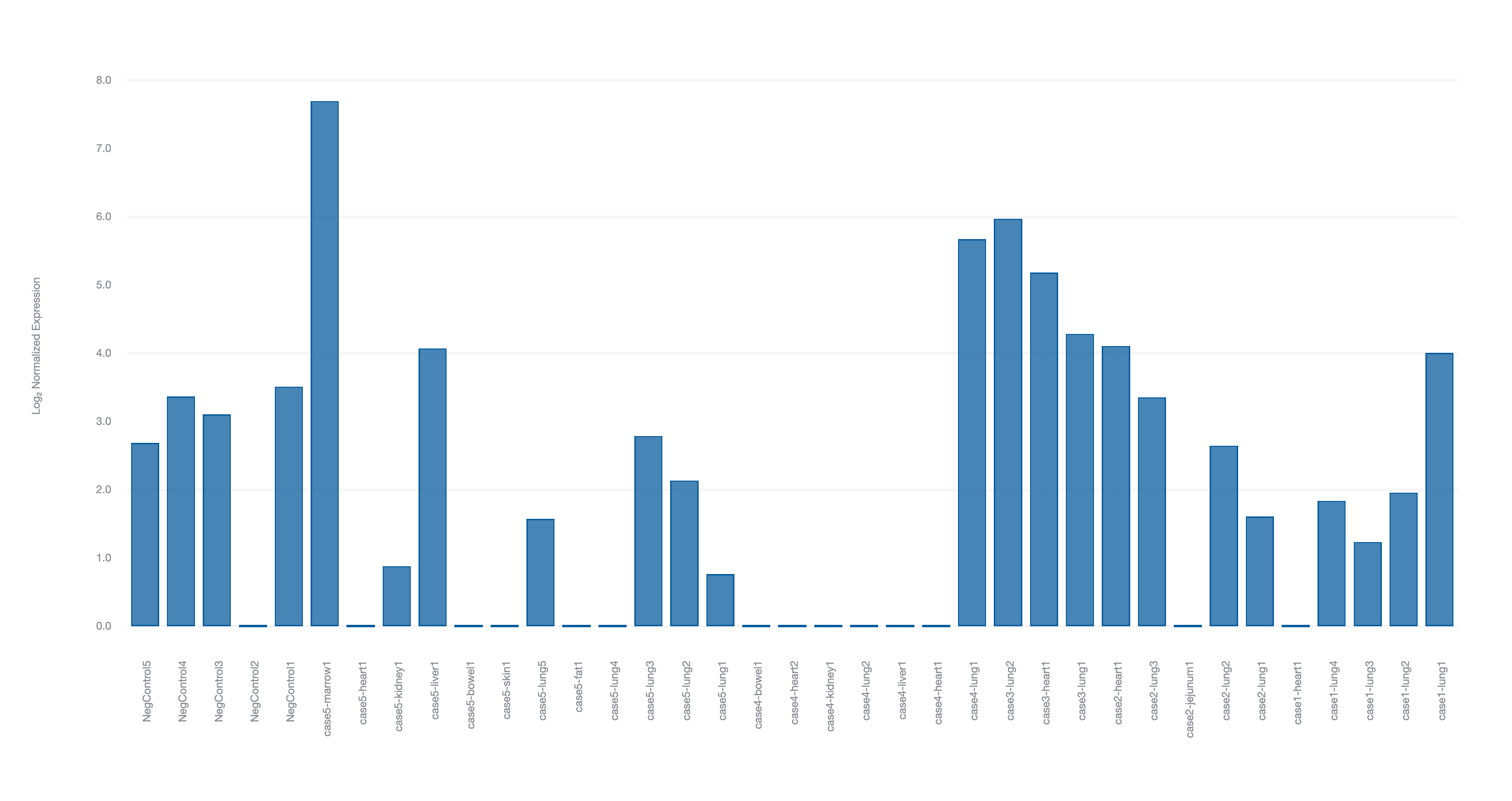

Supplement: Supplementary file 1 [file ijms-21-04662-s001.zip › Supp Fig S4 pad4 all tissues.png]

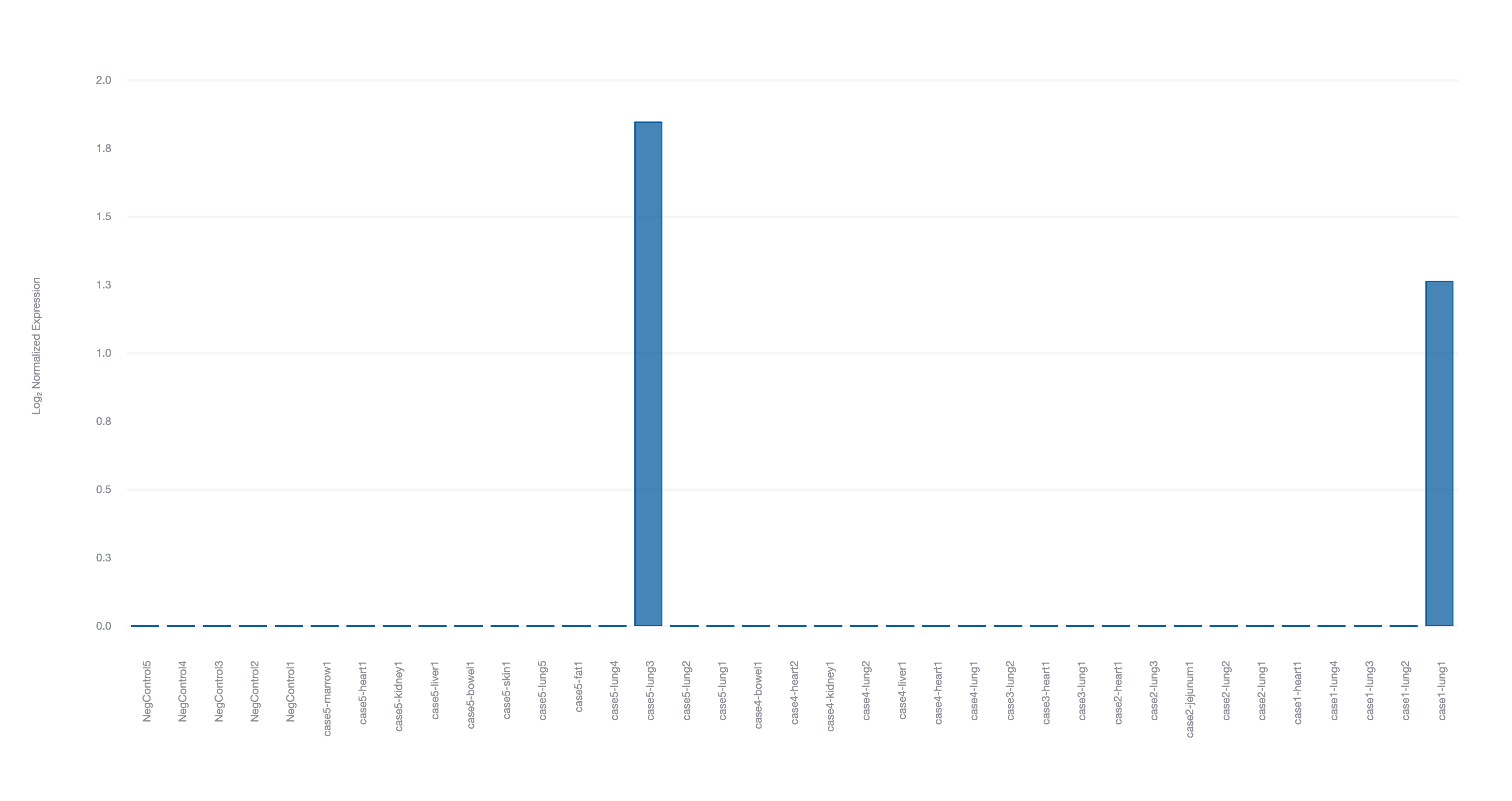

Supplement: Supplementary file 1 [file ijms-21-04662-s001.zip › Supp Fig S5 pad6 all tissues.png]
